# Retention Model of TaO/HfO_*x*_ and TaO/AlO_*x*_ RRAM with Self-Rectifying Switch Characteristics

**DOI:** 10.1186/s11671-017-2179-5

**Published:** 2017-06-13

**Authors:** Yu-De Lin, Pang-Shiu Chen, Heng-Yuan Lee, Yu-Sheng Chen, Sk. Ziaur Rahaman, Kan-Hsueh Tsai, Chien-Hua Hsu, Wei-Su Chen, Pei-Hua Wang, Ya-Chin King, Chrong Jung Lin

**Affiliations:** 1 Electronic and Optoelectronic System Research Laboratories, Industrial Technology Research Institute, Hsinchu, 310 Taiwan; 2 Microelectronics Laboratory, Institute of Electronics Engineering, National Tsing Hua University, Hsinchu, 310 Taiwan; 3 Department of Chemical and Materials Engineering, MingShin University of Science and Technology, Xinfeng, 304 Taiwan

**Keywords:** Retention, TaO/HfO_*x*_, TaO/AlO_*x*_, Self-rectifying, Resistive memory, Trapping-type

## Abstract

A retention behavior model for self-rectifying TaO/HfO_*x*_- and TaO/AlO_*x*_-based resistive random-access memory (RRAM) is proposed. Trapping-type RRAM can have a high resistance state (HRS) and a low resistance state (LRS); the degradation in a LRS is usually more severe than that in a HRS, because the LRS during the SET process is limited by the internal resistor layer. However, if TaO/AlO_*x*_ elements are stacked in layers, the LRS retention can be improved. The LRS retention time estimated by extrapolation method is more than 5 years at room temperature. Both TaO/HfO_*x*_- and TaO/AlO_*x*_-based RRAM structures have the same capping layer of TaO, and the activation energy levels of both types of structures are 0.38 eV. Moreover, the additional AlO_*x*_ switching layer of a TaO/AlO_*x*_ structure creates a higher O diffusion barrier that can substantially enhance retention, and the TaO/AlO_*x*_ structure also shows a quite stable LRS under biased conditions.

## Background

Because NAND flash technology is facing a scaling limit, vertical resistive random-access memory (VRRAM) designs with low film stacks, high manufacturing yields, and no cross-coupling problems are promising candidates for high-density memory applications [1–3]. The 1TnR architecture with three-dimensional (3D) vertical structure helps realize ultralow bit cost for highly compact dense arrays [4–6]. Several researchers have proposed operating RRAM at low current levels by changing the resistance switching mechanism from a filamentary-type to a defect-trapping-, vacancy-modulating-, or interface-type conducting path model [7–9]. However, the questions central to retention failures and the migration of oxygen vacancies are still unsolved [3, 10]. In some filamentary-type retention studies, many different models have been proposed to explain retention losses [11–13]. The change of switching mechanism also indicates a different direction that might improve retention [11]. Our previous studies have shown that TaO/HfO_*x*_ devices can show favorable nonlinearity values of approximately 40, endurance values exceeding 1000 cycles, and 85 °C data retention [6, 7]. Nevertheless, to obtain stable retention at such low operating current levels is still challenging. In this letter, a retention model is proposed to realize the retention loss in two different defect-trapping-type devices with the Arrhenius method. The extracted activation energy does not convincingly explain the retention improvement by the AlO_*x*_ layer. Even though the original was ambiguous, the most likely interpretation is that dense bonding facilitates retention.

## Methods

**Fig. 1:**
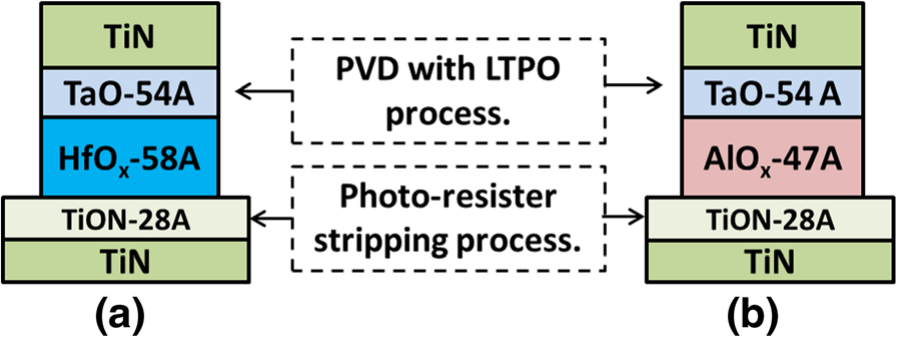
Cell schematic plots with thickness information for **a** TaO/HfO_*x*_ devices and **b** TaO/AlO_*x*_ devices. Both plots describe devices for which PVD deposited the TaO layers with LTPO processes, and the bottom TiON interfacial layers were formed by plasma oxidation during photoresist removal

In the fabrication of TaO/HfO_*x*_ and TaO/AlO_*x*_ devices for the present study, the bottom electrode (BE) is composed of TiN metal deposited by physical vapor deposition (PVD) on 8-in. thermal oxide/Si substrates. Each BE was patterned and etched with a conventional lithography and etching process. After each TiN BE had been etched with chlorine-based gas, the remaining photoresist (PR) and etching residues were removed using a remote plasma system that applied O_2_ and H_2_O at 180 °C. During the PR removal process, a thin oxidation layer of TiON was formed on the surface of each TiN BE. Then, resistive switching layers of HfO_*x*_ and AlO_*x*_ were prepared through atomic layer deposition (ALD) with HfCl_4_-H_2_O and TMA-H_2_O precursors, respectively. The two resistive elements HfO_*x*_ and AlO_*x*_ were deposited at 300 and 250 °C. On the top of resistive switching layers, the TaO layer was then deposited by PVD through low-temperature plasma oxidation (LTPO) [14]. This fabrication deposits Ta metal at an ultralow rate (0.2 Å/s). Stable plasma oxidation was performed with a mixture of Ar and O_2_ gases. This TaO layer served as an internal self-compliance resistance, which was relatively leaky compared with prior resistive switching films [7]. The top electrode was also PVD-TiN. The cross-sectional views and thickness information of the TaO/HfO_*x*_ and TaO/AlO_*x*_ memory devices are illustrated in Fig. 1a, b respectively. The film thickness of TaO/HfO_*x*_ was checked by transmission electron microscopy (not shown). After the cells had been patterned, the low-temperature oxide was deposited for passivation at 250 °C. Finally, a conventional back-end process was applied to finish the fabrication of contact and metal pad structures.

## Results and Discussion

**Fig. 2:**
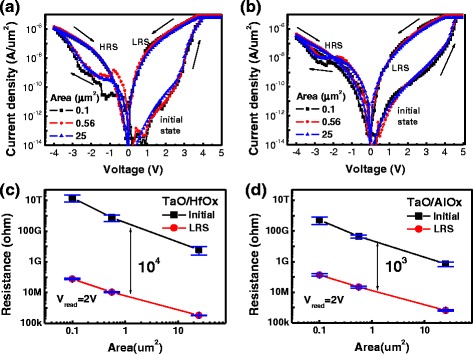
Current density with voltage plot of **a** TaO/HfO_*x*_ devices with different cell sizes. **b** TaO/AlO_*x*_ devices with different cell sizes. The resistance versus the area plot of **c** a TaO/HfO_*x*_ device and **d** a TaO/AlO_*x*_ device. Both plots contain the IRS and LRS with reading voltage = 2 V. Each data point provides the average of 10 devices and the corresponding standard deviation

The electrical measurements were performed with a HP4156C semiconductor parameter analyzer. The set and reset current density (*J*) versus voltage (*J*–*V*) curves of TaO/HfO_*x*_ and TaO/AlO_*x*_ devices are shown in Fig. 2a, b respectively. Both initial resistance states (*R*_initial_) of the TaO/HfO_*x*_ and TaO/AlO_*x*_ devices were HRS. The virgin memory devices were programmed to LRS with positive bias and were swept back. Then, each cell was switched from LRS to HRS by applied negative voltage. Both *J–V* plots contain three cell sizes, namely, 0.1, 0.56, and 25 μm^2^. In the *J–V* plots, all curves from devices with different areas resemble each other, which indicates both TaO/HfO_*x*_ and TaO/AlO_*x*_ devices had (i) the same current density in the initial state, (ii) similar set and reset voltages, and (iii) the same current density in LRS and HRS. Moreover, the constant current density property is clearly illustrated by the resistance versus area (*R*–*A*) plots in Fig. 2c, d. The strong area dependence in both *R*_initial_ and LRS can be observed by the control of current density. Regardless of the scale of cell area and compliance current, the same on/off resistance ratio is kept in both devices. This constant current density switch characteristic implies the memory cells are uniformly programmed or erased by the electrical field. These devices are considered to have trapping-type switching properties, which strongly relate to the modulation of vacancies [8]. In the case of trapping-type RRAM, no sharp current jump has been observed during the set process, but sharp current jumps have been commonly observed for filamentary-type RRAM. In the present research, different switching voltages were observed for the different switching layers with HfO_*x*_ or AlO_*x*_. The set voltage range of a TaO/AlO_*x*_ device is 4 to 4.5 V, which is larger than that of a TaO/HfO_*x*_ device (3 to 4 V). The reset voltage range of a TaO/AlO_*x*_ device is −1.5 to −2.5 V, which is larger than that of a TaO/HfO_*x*_ device (−0.5 to −1.5 V). An AlO_*x*_ system consumes more energy to complete the set and reset switches than a HfO_*x*_ system consumes. During the setting of switches, the switching layers HfO_*x*_ and AlO_*x*_ achieve soft breakdowns at voltages of approximately 3 and 3.5 V, respectively. In both types of devices, before filaments form in the switching layer, the current is limited by the internal resistance of the TaO layer. During the self-compliance process of trapping-type RRAM, excessive oxygen vacancies are generated inside the switching layer [7]. Those oxygen vacancies are recombined during the negative biasing reset process. Unlike filamentary-type RRAM, the HRS is always lower than the initial resistance state (IRS) after a reset operation [15–17]. To summarize, defect-trapping is a process that modulates vacancies through oxygen ion–vacancy recombination to control the resistance variation in the switching layer. Compared with a HfO_*x*_ switching layer, defect-trapping causes higher voltage and power in the AlO_*x*_ layer during both the setting and the resetting of a switch.

**Fig. 3:**
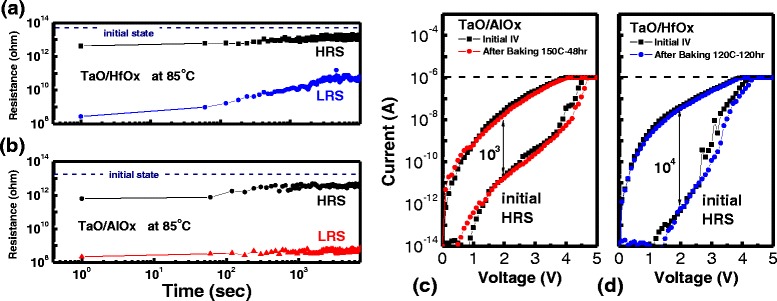
Plots of resistance variation versus time for **a** TaO/HfO_*x*_ and **b** TaO/AlO_*x*_ devices. Both plots contain HRS and LRS variation at reading voltage = 1 V in 85 °C. After the *I*–*V* sweeps of each virgin device had been set, the device was baked and then programmed to LRS again: **c** TaO/AlO_*x*_ (150 °C for 48 h); **d** TaO/HfO_*x*_ (120 °C for 120 h)

After the switching behavior had been investigated, the HRS and LRS retention behaviors of the trapping-type memory units were investigated. The plots of resistance variation versus time at 85 °C and 1 V for the TaO/HfO_*x*_ and TaO/AlO_*x*_ devices are shown in Fig. 3a, b. In both plots, the LRS variation is more pronounced than the HRS variation. The resistance stability of TaO/AlO_*x*_ is higher than that of TaO/HfO_*x*_. The figures illustrate that the HRSs tended to drift toward the IRSs for both types of devices; the IRSs are marked by dashed lines in Fig. 3a, b. The trend of resistance coming back to device’s virgin state is depicted in Fig. 3c for TaO/AlO_*x*_ and in Fig. 3d for TaO/HfO_*x*_. To realize this, both types of devices were initially programmed to LRS at room temperature, as shown in the *I–V* sweeps (black line). Then, the TaO/AlO_*x*_ and TaO/HfO_*x*_ devices were baked in ovens at 150 °C for 48 h and at 120 °C for 120 h, respectively. For both cases, the *I–V* sweep after having been baked was similar to the initial sweep. By this procedure, the LRSs of trapping-type devices were returned to the original states after time in a high-temperature environment. Unlike filament-type devices, which feature notable movement of oxygen atoms, trapping-type devices have pairs of oxygen ions and vacancies separated by short distances. The tendency of resistance drifting to the initial state is related to its original crystallinity, which is mainly controlled by the process temperature of ALD. As a result, the LRSs in both types of devices can be reset to HRSs (or IRSs) by negative bias or thermal energy. This property is different with filamentary RRAM.

**Fig. 4:**
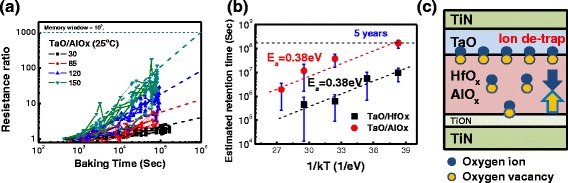
**a** Resistance variation ratio versus baking time for different temperatures in TaO/AlO_*x*_ devices. The average initial resistance was 179 MOhm with a reading voltage of 2 V, and the LRS resistance degradation rate was calculated by the linear fitting method in log(*R*_ratio_)–log(T) scale. **b** Estimated retention time (1000×) versus 1/kT plot. Each point contains data from 18 devices taken at a reading voltage of 2 V. The extracted activation energies were 0.38 eV in both the TaO/AlO_*x*_ and TaO/HfO_*x*_ devices. **c** Retention schematic diagram of different oxygen diffusion barriers in HfO_*x*_ or AlO_*x*_ with a TaO capping layer

In standard retention testing for nonvolatile memory, data retention is tested at both room temperature and at high temperature; devices must be able to retain data at both room temperature and at high temperature to be useful in real applications. Activation energy (*E*_a_) extraction by the Arrhenius method in the retention plot is a common method to evaluate data retention [18, 19]. As can be seen in Fig. 3a, the LRS variation is more pronounced than the HRS variation. Therefore, the resistance ratio (*R*_ratio_) of LRS versus baking time at temperatures ranging from 30 to 150 °C was analyzed. One example of retention time extraction from a TaO/AlO_*x*_ device is shown in Fig. 4a. The resistance degradation rate can be calculated by the slope of linear fitting in log(*R*_ratio_)-log(time) scale. By considering the maximum on/off resistance ratio of approximately 10^3^ for a TaO/AlO_*x*_ device, as shown in Fig. 3c, a retention time with 10^3^ times the LRS variation can be calculated. The estimated LRS data retention at measurement temperatures ranging from 30 to 150 °C is shown in Fig. 4b. Each data point represents information from more than 18 devices for both device types. In a TaO/AlO_*x*_ device, data retention is as high as 10^6^ s at 150 °C and 2 × 10^8^ s (approximately 5 years) at room temperature; those times are almost 10^1.5^ times longer than those of a TaO/HfO_*x*_ device. The most interesting point is that both TaO/HfO_*x*_ and TaO/AlO_*x*_ devices show the same *E*_a_ = 0.38 eV, as calculated from the extracted slope. The same *E*_a_ implies that both types of devices undergo similar chemical reactions in the LRS degradation process. This *E*_a_ is involved in all thermally activated kinetic processes, including the release of oxygen ions near TaO interfaces and the oxygen diffusion processes in AlO_*x*_ and HfO_*x*_ layers. However, the oxygen self-diffusion coefficients of HfO_*x*_ and AlO_*x*_ layers are different at high temperatures (>1000 °C); exact measurements can be found in the literature [20, 21]. The oxygen diffusion coefficient at low temperature (<200 °C) also depends on the thickness of HfO_*x*_ dielectrics [22]. If the diffusion processes in switching layers dominate the chemical reaction, then the *E*_a_ values should be different due to the different diffusion coefficients in HfO_*x*_ and AlO_*x*_ layers. Both types of devices in this work exhibited the same *E*_a_ = 0.38 eV; this was related to the fact that both types of devices had the same capping layer of TaO on the top of the switching layers. LRS degradation is a process of recombination of vacancies and ions, which means the TaO layer controls this chemical reaction and most of the vacancies are crowded near the interface between the TaO and the switching layer. Those vacancies prefer to stay on the TaO/switching layer interface; this phenomenon could be supported by the thermodynamic stability point of view, as reported by Zhong et al. [23]. In their simulation of TiN/Ta/HfO_*x*_/TiN stacks, the oxygen ions preferred to stay on the Ta/HfO_*x*_ interface because a low energy difference existed between Ta and HfO_*x*_ [23]. In their simulation, as in the present experiments, the TaO resistive layer trapped most of the oxygen ions and dominated this vacancy recombination process. LRS degradation is schematized in Fig. 4c. The oxygen ions return to the previous thermal equilibrium state during the baking process, which results in retention loss. Differences can be noted between the Ta/HfO_*x*_ device as proposed by Zhong et al. and the TaO/HfO_*x*_ device in this study, but in both studies, the TaO layer was formed by several cycles of metal Ta deposition and LTPO processes [14]. Because of the LTPO process, the metal-rich TaO/HfO_*x*_ interface can be considered as an oxygen ion reservoir. During the recombination process of oxygen ions and vacancies, the atom packing density plays an essential role. The superior LRS retention properties obtained in the AlO_*x*_ switching layer could be explained by the high atomic density of the AlO_*x*_ layer. It is well known that the bond length of Al–O is shorter than that of Hf–O [24, 25]. The short bond in the AlO_*x*_ reduces the oxygen ion mobility due to high coulomb interaction, which results in a high oxygen vacancy diffusion barrier. This barrier causes retention time to be longer in a TaO/AlO_*x*_ device than in a TaO/HfO_*x*_ one.

**Fig. 5:**
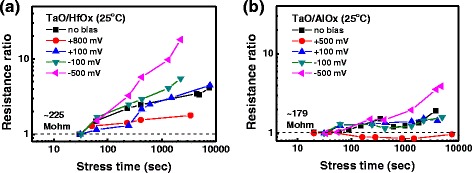
On-bias resistance ratio versus stress time for **a** TaO/HfO_*x*_ and **b** TaO/AlO_*x*_ devices at room temperature

In addition, the retention loss model of a filamentary-type device is different from that of a defect-trapping-type device. The retention behavior for filamentary-type RRAM is related to filament rupture, and the vacancy diffusion direction is lateral [11, 19, 24]. In defect-trapping RRAM, the defect diffusion direction is longitudinal, which is parallel to the external electric field. Therefore, the retention behavior can be affected by the biasing direction and magnitude. Figure 5a, b shows the on-bias retention through the resistance ratio for the two devices. The resistance ratio is defined as the resistance of the stress device to the resistance of the LRS. A positive bias can help to maintain the LRS, but a negative bias accelerates the degradation process. Those on-bias properties could be explained by the interaction between the localized field of pairs of oxygen ions and vacancies and the external electrical field. If the direction of external field is the same as the set direction (positive), it extends retention time; if the external field is in the reset direction (negative), it causes degradation. In a low electric field with ±100 mV, the on-bias degradation is the same as the no-bias degradation in both types of devices. This ±100 mV bias might be covered by the band offsets of TiON-HfO_*x*_, TiON-AlO_*x*_, and TiN-TaO junctions. A TaO/AlO_*x*_ device under a high positive bias of 500 mV shows no obvious degradation.

## Conclusions

In summary, we compared two types of self-rectified RRAM devices through their switch characteristics and analyzed their retention behaviors. The TaO/AlO_*x*_ device showed a higher switching voltage and a more robust LRS thermal stability than the TaO/HfO_*x*_ device did. The benefit of robust retention from the AlO_*x*_ switching layer is due to the high oxygen diffusion barrier rather than activation energy. The activation energy of retention loss is related to the ion de-trap process in the TaO resistive layer. The high atomic density of AlO_*x*_ film may improve LRS retention. A retention loss schematic model has been proposed and the on-bias retention results supported this model. This model could be beneficial for the development of low-current, long-retention, self-rectifying RRAM devices for future high-density memory applications.
